# A comparison of death recording by health centres and civil registration in South Africans receiving antiretroviral treatment

**DOI:** 10.7448/IAS.18.1.20628

**Published:** 2015-12-16

**Authors:** Leigh F Johnson, Rob E Dorrington, Ria Laubscher, Christopher J Hoffmann, Robin Wood, Matthew P Fox, Morna Cornell, Michael Schomaker, Hans Prozesky, Frank Tanser, Mary-Ann Davies, Andrew Boulle, for the International Epidemiologic Databases to Evaluate AIDS Southern Africa (IeDEA-SA) Collaboration

**Affiliations:** 1 Centre for Infectious Disease Epidemiology and Research, University of Cape Town, Cape Town, South Africa; 2 Centre for Actuarial Research, University of Cape Town, Cape Town, South Africa; 3 Burden of Disease Research Unit, South African Medical Research Council, Cape Town, South Africa; 4 Aurum Institute, Johannesburg, South Africa; 5 Division of Infectious Diseases, Johns Hopkins School of Medicine, Baltimore, MD, USA; 6 Desmond Tutu HIV Centre, University of Cape Town, Cape Town, South Africa; 7 Center for Global Health and Development, Boston University, Boston, MA, USA; 8 Health Economics and Epidemiology Research Office, Department of Medicine, Faculty of Health Sciences, University of the Witwatersrand, Johannesburg, South Africa; 9 Division of Infectious Diseases, Department of Medicine, Tygerberg Academic Hospital, University of Stellenbosch, Cape Town, South Africa; 10 Wellcome Trust Africa Centre for Health and Population Studies, University of KwaZulu-Natal, Mtubatuba, South Africa; 11 School of Nursing and Public Health, University of KwaZulu-Natal, Durban, South Africa

**Keywords:** antiretroviral therapy, HIV, vital statistics registration, South Africa

## Abstract

**Introduction:**

There is uncertainty regarding the completeness of death recording by civil registration and by health centres in South Africa. This paper aims to compare death recording by the two systems, in cohorts of South African patients receiving antiretroviral treatment (ART).

**Methods:**

Completeness of death recording was estimated using a capture–recapture approach. Six ART programmes linked their patient record systems to the vital registration system using civil identity document (ID) numbers and provided data comparing the outcomes recorded in patient files and in the vital registration. Patients were excluded if they had missing/invalid IDs or had transferred to other ART programmes.

**Results:**

After exclusions, 91,548 patient records were included. Of deaths recorded in patients files after 2003, 94.0% (95% CI: 93.3–94.6%) were recorded by civil registration, with completeness being significantly higher in urban areas, older adults and females. Of deaths recorded by civil registration after 2003, only 35.0% (95% CI: 34.2–35.8%) were recorded in patient files, with this proportion dropping from 60% in 2004–2005 to 30% in 2010 and subsequent years. Recording of deaths in patient files was significantly higher in children and in locations within 50 km of the health centre. When the information from the two systems was combined, an estimated 96.2% of all deaths were recorded (93.5% in children and 96.2% in adults).

**Conclusions:**

South Africa's civil registration system has achieved a high level of completeness in the recording of mortality. However, the fraction of deaths recorded by health centres is low and information from patient records is insufficient by itself to evaluate levels and predictors of ART patient mortality. Previously documented improvements in ART mortality over time may be biased if based only on data from patient records.

## Introduction

Antiretroviral treatment (ART) has had a significant impact on AIDS mortality in developing countries [1–4]. However, most of what is known regarding mortality after ART initiation in developing countries derives from patient record systems in health centres and has not been validated by data from vital registration systems. Patient record systems may miss substantial numbers of deaths, leading to underestimation of mortality [5]. As clinic volumes increase, capacity to ascertain reasons for loss from care and deaths may be reduced, leading to further limitations on using patient record systems to understand mortality among individuals receiving ART.

Vital registration systems have an important role to play in the ascertainment of mortality levels generally, including that in ART patients [6–8]. However, in many developing countries vital registration is too incomplete to permit the ascertainment of vital status with a high degree of confidence [9]. For incomplete vital registration data to be useful, it is essential that completeness of registration be reliably estimated, so that recorded death data can be appropriately adjusted when calculating mortality rates. The completeness of adult death reporting at a population level is usually estimated by death distribution methods (DDMs), which attempt to estimate the true number of deaths based on age-specific changes in population size between censuses. These methods work well, provided the assumptions hold. However, the methods may be biased due to age misreporting, migration (if not accounted for) and other factors, which can lead to estimated levels of completeness that differ substantially from true completeness levels [10,11]. Given this lack of precision, it has been suggested that alternative methods, such as capture-recapture, should be considered in estimating the completeness of vital registration of mortality [10].

South Africa is an upper-middle-income country in which a high proportion of deaths are reported through the vital registration system. DDM-derived estimates suggest that the completeness of adult death recording increased from about 84% in the 1996–2001 period [12] to around 93% in the period after 2001 [13–15]. However, there have been few attempts to verify these estimates of completeness using alternative methodologies. In addition, DDMs do not estimate the completeness of death reporting in children. There is substantial uncertainty regarding the fraction of child deaths that have been reported, which is a major obstacle in measuring South Africa's progress towards meeting Millennium Development Goal 4.

Given the need for both better assessment of outcomes of ART programmes and better understanding of the completeness of South Africa's vital registration system, we aim to use data from cohorts of South African ART patients to determine the relative performance of the patient record and vital registration systems in identifying patient deaths.

## Methods

Patients receiving ART in South Africa were identified through the International epidemiologic Databases to Evaluate AIDS Southern Africa (IeDEA-SA) collaboration, a network of ART programmes from across Southern Africa [16]. For the purpose of this analysis, patients were included only if they had recorded civil identity document (ID) numbers. Recording of patient IDs has improved over time, with over two thirds of patients having recorded IDs in recent years [6,7]. Six ART programmes contributed data: the Khayelitsha, Gugulethu and Tygerberg programmes (Western Cape province); the McCord and Hlabisa programmes (KwaZulu-Natal); and the Themba Lethu programme (Gauteng). In the early stages of their operation, all programmes attempted to contact patients who missed visits, but over time, growing patient numbers made this increasingly difficult, and only the Tygerberg and Gugulethu programmes have continued active follow-up. Participating ART programmes recorded the patient deaths of which they were aware in their patient record systems.

Civil registration of deaths occurred through the Department of Home Affairs, which maintains a National Population Register of the vital status of all individuals with IDs. At fixed times, participating ART programmes submitted a list of civil ID numbers of patients to the South African Medical Research Council (MRC), which maintains a monthly updated database of all deaths recorded in South Africa with civil IDs, obtained from the Department of Home Affairs [17]. The MRC returned to each ART programme information on the vital status of each patient, including information on the date of death and place of death if a death was recorded. Each ART programme then provided anonymized data to the IeDEA-SA data centre, including the original vital status recorded in patient files and the vital status information from the National Population Register. All IeDEA-SA programmes obtained ethical approval from local institutions before linking patient records to the MRC database and before contributing anonymized data to the collaborative analysis. In addition, the collaboration obtained approval from the University of Cape Town Human Research Ethics Committee to receive and analyze these data.

Patients were excluded from all analyses if they had an invalid ID number or a duplicate ID (the same ID number assigned to more than one patient), or if they were recorded as having transferred to another ART programme in patient files (since ART programmes were not expected to determine the vital status of patients who were known to have transferred). For the purpose of the analysis of completeness of the civil registration system, deaths recorded by health centres were included only if they occurred between 1 January 2004 (the approximate date of the start of the public-sector ART programme in South Africa) and 365 days prior to the date on which the linkage to the National Population Register occurred (to allow sufficient time for late reporting of deaths). Similarly, for the purpose of the analysis of the completeness of death recording by health centres, deaths recorded through the civil registration system were included only if they occurred between 1 January 2004 and 365 days prior to the date on which the last patient visit/outcome was recorded. The analysis closure dates and vital registry linkage dates differed between ART programmes. Although most ART programmes linked their patient records to the National Population Register on multiple dates, this analysis is limited to the data that were available at the time that the cohorts performed their *first* linkage, due to concern that some programmes may have updated their patient files with the vital registry data after the first linkage occurred, thus compromising the independence of the two data sources.

Two multivariate logistic regression models were fitted to assess the predictors of death recording by the two systems, and the best models in both cases were chosen based on the Bayesian Information Criterion (BIC) [18]. Variables included age, sex, year, ART programme, programme location and location of death (deaths were defined as having occurred locally if the place of death recorded on the National Population Register was within 50 km of the health centre at which the patient received ART).

As in other studies of death reporting in developing countries [19–24], we used a capture-recapture approach: deaths that are identified through one recording system (the “capture” step) are matched against deaths identified through a second recording system (the “recapture” step) and the number of deaths that are identified by neither system is estimated, assuming the fractions recorded are statistically independent [25]. The capture-recapture method incorporated the covariate effects identified as relevant by BIC in the multivariate logistic regression models [26,27].

As there is a need for age- and sex-specific correction factors that can be applied to vital registration statistics, we fitted different models to predict *c*(*x*, *g*), the fraction of deaths occurring at age *x*, in individuals of sex *g*, that are recorded through the vital registration system (further detail is provided in the Supplementary File). The best logistic model, based on the BIC, was of the form

$$c(x,g)=\frac{1}{1+\exp\left(-\alpha -\beta g\exp\left(-2{\left(\ln\left(\frac{x+1}{25+1}\right)\right)}^{2}\right)-\gamma \ln(x+1)-\delta \exp(-x)\right)},$$

where *α* is a constant term, *β* represents the effect of male sex at young ages (*g*=0 for females and 1 for males),*γ* represents the effect of age after infancy and *δ* represents the effect of age in infants. The predicted completeness of vital registration for the country as a whole, in 2010, was calculated by applying the resulting correction factors (1/*c*(*x*, *g*)) to the recorded numbers of deaths in South Africa in 2010 [28] and dividing the total number of recorded deaths in 2010 by the corrected total. All statistical analyses were performed using STATA version 13 (StataCorp, College Station, TX, USA).

## Results

The first linkages to the National Population Register were performed in March 2008 (Khayelitsha), September 2011 (Hlabisa, McCord, Themba Lethu) and February 2015 (Gugulethu, Tygerberg). A total of 104,143 patient records were linked to the National Population Register, but 663 records could not be linked due to invalid IDs. After excluding duplicate IDs (1929) and patients who had transferred out of care (10,003), a total of 91,548 patients remained. Out of this total, 5346 individuals were recorded as dead by both health centres and vital registration, with one or both sources recording the date of death after the start of 2004. Table 1 shows the extent of the agreement between the two recorded death dates in these cases. In 79.4% of cases the two dates were within 30 days of one another, but in 4.3% of cases the two dates were more than 365 days apart. The recorded date of death was earlier on average in the vital registration system.

**Table 1:** Consistency in recording of date of death

Date of death on National Population Register	*n*	%
>365 days prior to date of death on patient record	189	3.5
31–365 days prior to date of death on patient record	513	9.6
1–30 days prior to date of death on patient record	1140	21.3
Same as date of death on patient record	2100	39.3
1–30 days after date of death on patient record	1005	18.8
31–365 days after date of death on patient record	356	6.7
>365 days after date of death on patient record	43	0.8
Total	5346	

A total of 5207 deaths were recorded by health centres in the period between 1 January 2004 and the date 1 year prior to the date of death registry linkage. Of these, 94.0% (95% CI: 93.3–94.6%) were also recorded by the National Population Register. Recording through the vital registration system was more frequent in women, older adults and urban ART programmes (Tables 2 and 3). We could not show an effect of the province of the ART programme (after controlling for urban/rural location) or a change in completeness over time when a linear time trend was included in the model (*p*=0.14).

**Table 2:** Completeness of death reporting

	Deaths recorded in patient files		Deaths recorded in death register	
		
	*n*	% recorded in death register	*n*	% recorded in patient files
Year of death				
2004	98	90.8 (83.3–95.7)	155	58.7 (50.5–66.5)
2005	414	97.1 (95.0–98.5)	681	60.5 (56.7–64.2)
2006	604	93.5 (91.3–95.4)	1409	40.5 (37.9–43.1)
2007	874	92.2 (90.2–93.9)	2572	32.8 (31.0–34.7)
2008	1008	93.6 (91.9–95.0)	3033	32.7 (31.1–34.4)
2009	1229	94.1 (92.7–95.4)	3321	34.2 (32.6–35.9)
2010	799	94.4 (92.5–95.9)	2297	29.0 (27.1–30.9)
2011–2014	181	97.8 (94.4–99.4)	447	34.0 (29.6–38.6)
Sex				
Male	2376	93.0 (91.9–94.0)	6228	35.2 (34.0–36.4)
Female	2831	94.8 (93.9–95.6)	7687	34.8 (33.7–35.8)
Age at death				
< 15	117	77.8 (69.2–84.9)	133	67.7 (59.0–75.5)
15–29	909	93.3 (91.5–94.8)	2497	34.2 (32.3–36.1)
30–39	2023	94.2 (93.1–95.1)	5535	34.1 (32.9–35.4)
40–49	1385	94.1 (92.7–95.3)	3632	35.6 (34.1–37.2)
50 +	773	96.5 (95.0–97.7)	2118	34.8 (32.8–36.9)
Programme locality				
Urban	3767	95.4 (94.6–96.0)	10,423	34.2 (33.3–35.1)
Rural	1440	90.3 (88.7–91.8)	3492	37.3 (35.7–39.0)
Programme province				
Gauteng	2435	95.4 (94.5–96.2)	8242	28.2 (27.3–29.2)
KwaZulu-Natal	2013	91.8 (90.5–92.9)	4376	42.5 (41.0–43.9)
Western Cape	759	95.3 (93.5–96.7)	1297	52.5 (49.7–55.3)
Place of death^a^				
< 50 km from clinic	–	–	8473	43.1 (42.0–44.2)
≥ 50 km from clinic	–	–	5442	22.3 (21.2–23.4)
Total	5207	94.0 (93.3–94.6)	13,915	35.0 (34.2–35.8)

a Place of death was recorded only for those deaths recorded on the death register.

**Table 3:** Multivariate analysis of factors associated with recording of death

	Odds ratio (95% CI)	*p*
Recording of death by civil registration system		
Male sex	0.66 (0.52–0.83)	<0.001
Urban location	2.05 (1.62–2.59)	<0.001
Per 10-year increase in age	1.36 (1.23–1.50)	<0.001
Recording of death by health centres^a^		
Age <15	3.02 (2.05–4.44)	<0.001
Per year of increase in date of death	0.84 (0.82–0.86)	<0.001
Place of death <50 km from clinic	2.32 (2.13–2.52)	<0.001

a Controlling for differences between cohorts.

A total of 13,915 deaths were recorded on the National Population Register between 1 January 2004 and the date 1 year prior to the last patient observation in each ART programme. Of these, only 35.0% (95% CI: 34.2–35.8%) were recorded in patient files, with recording in patient files being more frequent for children, individuals dying in earlier years (2004–2005) and individuals dying within 50 km of the clinic at which they were treated (Table 2). In the multivariate analysis, differences between programmes were significant (results not shown) and the effects of age, year and location of death remained significant after controlling for other factors (Table 3).

The multivariate models in Table 3 were used to calculate the probability of a death being recorded by either of the death recording methods, for each individual, and the capture–recapture method was applied using these estimated probabilities [26,27]. Based on this approach, the estimated fraction of all deaths that are recorded through either system is 96.2%, with the proportion being marginally higher when comparing the population aged 15 and older to children under the age of 15 (96.2% vs. 93.5%).

**Figure 1:**
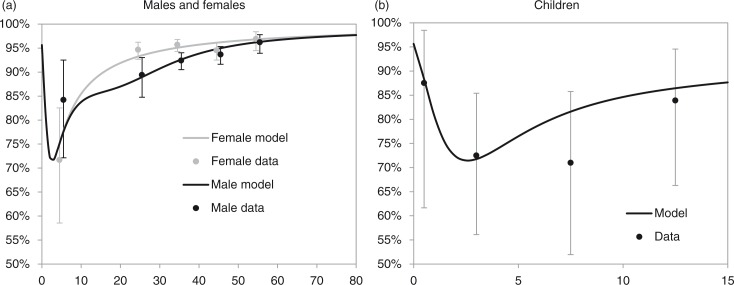
Fraction of deaths recorded by civil registration. In panel (a), data plotted at ages 5, 25, 35, 45 and 55 represent the average proportions over the 0–14, 15–29, 30–39, 40–49 and 50+ age groups, respectively. In panel (b), data plotted at ages 0.5, 3.0, 7.5 and 12.5 represent the average proportions over the <1, 1–4, 5–9 and 10–14 age groups, respectively. Model estimates shown in panel (b) are the averages of the estimates for males and females. For more detail regarding the statistical model parameters, see the Supplementary File.

Age and sex differences in vital registration completeness are shown in Figure 1. Differences between males and females were most pronounced in young adults (Figure 1a). Although completeness tended to decline with younger age, this trend was reversed in infants (Figure 1b). The statistical model fitted to the data estimates that the average completeness, weighted according to the age and sex profile of South African deaths in 2010, is 94.4% (87.0% in children aged <15 and 95.3% for ages 15 +).

In a few cases (0.23% of 56,924 in which the patient record indicated that the patient was alive), the National Population Register suggested that the patient had died more than a year before the last recorded date of clinic attendance.

## Discussion

This analysis suggests that by combining information from patient records and the vital registration system, South African ART programmes are able to identify more than 95% of deaths occurring in their programmes. This is comparable to – if not better than – levels achieved by ART programmes in high-income settings [29]. However, information from patient record systems is not sufficient by itself to obtain reliable estimates of the levels and predictors of mortality. It is particularly concerning that the proportion of deaths recorded in patient files has dropped from around 60% in the mid-2000s to around 30% in recent years. This reflects the increasing administrative pressures faced by public-sector ART programmes as they have expanded in size, and the attendant challenges in maintaining accurate records and following up on patients who miss visits. Although a number of ART programmes in developing countries have reported a trend towards lower rates of mortality over time [30–33], our findings suggest that much of the apparent mortality decline could be due to deterioration in death recording. Similarly, apparent increases over time in rates of loss to follow-up could be partially attributed to increasing rates of unrecorded mortality.

Recording of deaths in patient files was substantially higher in children than in adults, and consequently the need for vital registry linkage is less critical in children than in adults. This is probably because children have identified caregivers who can bring the child to the clinic if they are sick, and who can be contacted if the child does not return to the clinic. However, given that only a small proportion of children have recorded ID numbers, it is difficult to generalize from these findings. As might be expected, recording of deaths in patient files was also substantially higher when the death occurred locally than when the place of death was more than 50 km from the health centre. This is pertinent as almost 40% of all deaths did not occur locally; South Africa's population is highly mobile, and severely ill individuals often return to their ancestral homes [34].

This analysis suggests that approximately 95% of adult deaths with IDs in South Africa are recorded through the vital registration system, which is similar to recent estimates obtained using DDMs [13–15] and a 94% completeness among individuals with IDs in a demographic surveillance site [35]. Although this consistency with DDM estimates is reassuring, this analysis raises concern about one of the central assumptions of DDMs, namely that the completeness of death reporting is constant with respect to age in adults [11]. Our results suggest that completeness tends to increase with respect to adult age, consistent with age patterns of completeness observed in Thailand [21] and the Philippines [23]. Simulations suggest that if the completeness of death registration is low and increases steeply in relation to age, DDMs could substantially over-estimate the true level of completeness [11]. There is thus a need for extensions to existing DDMs to correct for this bias.

Vital registration of child deaths is generally believed to be less complete than that of adult deaths [10]. Although the number of child deaths in our analysis is too small to permit firm conclusions, it is interesting to note that the results suggest a relatively high proportion of infant deaths are recorded by the vital registration system, when compared to deaths in older children. Most other capture-recapture studies have found the opposite, with death recording being relatively poor in infants and neonates [19,20,22,23], although there have been exceptions [21]. Indirect methods for estimating child mortality in South Africa have confirmed the relatively high completeness of infant death reporting by the vital registration system [14,36]. Infant deaths may be more likely to be recorded because in South Africa a high proportion of these deaths occur in health facilities, and programmes such as the Child Healthcare Problem Identification Programme have been set up specifically to improve the monitoring of these facility-based deaths [17,37].

Although this analysis suggests that the completeness of death reporting is lower in males than in females, closer investigation reveals that most of this sex differential is concentrated in the young adult ages. This may be because young men have fewer family ties, and difficulties in identifying next of kin may hinder formal notification of deaths. Deaths in young women may be more likely to be registered if they are receiving child support grants, particularly if the caregivers of their orphans wish to register for foster care grants.

Although this analysis provides important insights into the factors affecting the completeness of vital registration in South Africa, some limitations need to be considered when assessing the generalizability of these results to the country as a whole. Patients attending public-sector services are generally of lower socioeconomic status, and HIV infection in South Africa tends to be associated with lower educational attainment and migrancy [38–40]. All of these factors might be associated with lower completeness of vital registration [35]. On the other hand, this analysis was limited to patients who had ID numbers; foreign nationals without South African IDs (who account for a substantial proportion of ART patients in some centres [41]) were excluded. Although patients with and without IDs have similar clinical and demographic characteristics [42], deaths in individuals without IDs might be less likely to get recorded by the vital registration system [35]. In addition, the ART programmes participating in this analysis were located in only three of South Africa's nine provinces, and there was a bias towards programmes in urban areas.

The finding that 35% of known deaths were recorded in patient files may not be generalizable to other settings. The programmes that are part of the IeDEA-SA collaboration tend to be better-resourced than other public-sector ART programmes, with most programmes having support from academic and non-governmental organization partners. In a non-research setting, another South African study found that only 16% of deaths recorded by the National Population Register were recorded in patient files [43]. In other African countries in which tracing studies have been conducted to obtain adjusted mortality estimates, crude mortality rates range from 18 to 70% of the corrected mortality estimates [44–48]. This suggests that the fraction of deaths that are recorded in patient files differs substantially between settings, depending on factors such as the resources available for tracking patients who miss visits, the patient mix (pre-ART vs. ART-experienced) and the levels of out-migration in the population.

Although capture-recapture methods are often used to assess the completeness of mortality and morbidity recording in developing countries [49], these methods have limitations. Most importantly, the methods assume that the recording of events in the capture and recapture steps is statistically independent; if this assumption is violated and there is strong positive dependence, the true total number of events may be substantially under-estimated (i.e. completeness over-estimated). This bias can be reduced by controlling for variables that account for the dependence [26,27], as we have done. However, to the extent that the dependence is due to unobserved covariates that cannot be controlled for, some bias is likely to remain. An alternative approach to limit bias is to collect data from a third source. In one such study from the Philippines, recording of deaths by the civil registration system was found to be strongly positively dependent on recording of deaths by health centres when compared against a third source (church records) [23]. However, it is not clear how much of the dependence would have been removed by controlling for covariates such as those considered in the present analysis.

Another capture-recapture assumption is that individuals in the two recording systems can be perfectly matched and that there are no “false-positive” records. The South African ID number is relatively unambiguous when compared to matching methods based on variables such as name, age and place of death [35]. However, there were a few cases of duplicate IDs, which might have been due to multiple patient files being opened for the same individual. There were also a few cases (0.23% of 56,924) where patient records indicated that the patient was alive but the National Population Register suggested that the patient had died more than a year before the last recorded date of clinic attendance; this could possibly be due to ID fraud (patients using the IDs of deceased individuals) or errors in the recording of ID numbers. Alternatively, these discrepancies may be due to errors in the recording of dates; Table 1 suggests that there is frequent inconsistency in the date of death recorded, and there may be similar errors in the recording of patient visit dates.

## Conclusions

Despite its limitations, civil registration remains the best tool for monitoring the mortality due to HIV and other causes in many developing countries [9]. By itself, the mortality data from patient record systems cannot be regarded as sufficient for monitoring purposes, or for assessing the predictors of mortality. In countries where the coverage of vital registration remains low, efforts to improve vital registration systems should be prioritized, and in clinical monitoring systems where it is not already in place, linkage to vital registration systems should be established. Tracing of a sample of patients who are considered lost to follow-up may also yield improved estimates of mortality in situations where linkage to vital registration is not feasible [44–46].

## Supplementary Material

A comparison of death recording by health centres and civil registration in South Africans receiving antiretroviral treatment

## References

[1] UNAIDS (2013). Global report: UNAIDS report on the global AIDS epidemic 2013.

[2] Mahy M, Stover J, Stanecki K, Stoneburner R, Tassie JM (2010). Estimating the impact of antiretroviral therapy: regional and global estimates of life-years gained among adults. Sex Transm Infect.

[3] April MD, Wood R, Berkowitz BK, Paltiel AD, Anglaret X, Losina E (2014). The survival benefits of antiretroviral therapy in South Africa. J Infect Dis.

[4] Bendavid E, Bhattacharya J (2009). The President's Emergency Plan for AIDS Relief in Africa: an evaluation of outcomes. Ann Int Med.

[5] Brinkhof MW, Pujades-Rodriguez M, Egger M (2009). Mortality of patients lost to follow-up in antiretroviral treatment programmes in resource-limited settings: systematic review and meta-analysis. PLoS One.

[6] Boulle A, Van Cutsem G, Hilderbrand K, Cragg C, Abrahams M, Mathee S (2010). Seven year experience of a primary care antiretroviral treatment programme in Khayelitsha, South Africa. AIDS.

[7] Fox MP, Brennan A, Maskew M, Macphail P, Sanne I (2010). Using vital registration data to update mortality among patients lost to follow-up from ART programmes: evidence from the Themba Lethu Clinic, South Africa. Trop Med Int Health.

[8] Cornell M, Schomaker M, Garone D, Giddy J, Hoffmann CJ, Lessells R (2012). Gender differences in survival among adult patients starting antiretroviral therapy in South Africa: a multicentre cohort study. PLoS Med.

[9] Setel PW, Macfarlane SB, Szreter S, Mikkelsen L, Jha P, Stout S (2007). A scandal of invisibility: making everyone count by counting everyone. Lancet.

[10] Murray CJ, Rajaratnam JK, Marcus J, Laakso T, Lopez AD (2010). What can we conclude from death registration? Improved methods for evaluating completeness. PLoS Med.

[11] Hill K, You D, Choi Y (2009). Death distribution methods for estimating adult mortality: sensitivity analysis with simulated data error. Demogr Res.

[12] Dorrington RE, Moultrie TA, Timæus IM (2004). Estimation of mortality using the South African Census 2001 data.

[13] Statistics South Africa (2014). Mortality and causes of death in South Africa, 2011: findings from death notification.

[14] Dorrington R, Bradshaw D, Laubscher R (2014). Rapid mortality surveillance report 2012.

[15] 
Dorrington RE, Bradshaw D (2011). Maternal mortality in South Africa: lessons from a case study in the use of deaths reported by households in censuses and surveys. J Popul Res.

[16] Egger M, Ekouevi DK, Williams C, Lyamuya RE, Mukumbi H, Braitstein P (2012). Cohort profile: the international epidemiological databases to evaluate AIDS (IeDEA) in sub-Saharan Africa. Int J Epidemiol.

[17] Joubert J, Rao C, Bradshaw D, Dorrington RE, Vos T, Lopez AD (2012). Characteristics, availability and uses of vital registration and other mortality data sources in post-democracy South Africa. Glob Health Action.

[18] Schwarz G (1978). Estimating the dimension of a model. Ann Stat.

[19] Yang G, Hu J, Rao KQ, Ma J, Rao C, Lopez AD (2005). Mortality registration and surveillance in China: history, current situation and challenges. Popul Health Metr.

[20] Eisele TP, Lindblade KA, Rosen DH, Odhiambo F, Vulule JM, Slutsker L (2003). Evaluating the completeness of demographic surveillance of children less than five years old in western Kenya: a capture-recapture approach. Am J Trop Med Hyg.

[21] Vapattanawong P, Prasartkul P (2011). Under-registration of deaths in Thailand in 2005–2006: results of cross-matching data from two sources. Bull World Health Organ.

[22] Diallo DA, Habluetzel A, Esposito F, Cousens SN (1996). Comparison of two methods for assessing child mortality in areas without comprehensive registration systems. Trans R Soc Trop Med Hyg.

[23] Carter KL, Williams G, Tallo V, Sanvictores D, Madera H, Riley I (2011). Capture-recapture analysis of all-cause mortality data in Bohol, Philippines. Popul Health Metr.

[24] El-Shalakani M (1985). Estimating the completeness of births and deaths registration in Egypt through dual record systems. Genus.

[25] Tilling K (2001). Capture-recapture methods – useful or misleading?. Int J Epidemiol.

[26] Tilling K, Sterne JA (1999). Capture-recapture models including covariate effects. Am J Epidemiol.

[27] Alho JM (1990). Logistic regression in capture-recapture models. Biometrics.

[28] Statistics South Africa (2014). Mortality and causes of death in South Africa, 2013: findings from death notification.

[29] May MT, Hogg RS, Justice AC, Shepherd BE, Costagliola D, Ledergerber B (2012). Heterogeneity in outcomes of treated HIV-positive patients in Europe and North America: relation with patient and cohort characteristics. Int J Epidemiol.

[30] Nsanzimana S, Remera E, Kanters S, Chan K, Forrest JI, Ford N (2015). Life expectancy among HIV-positive patients in Rwanda: a retrospective observational cohort study. Lancet Glob Health.

[31] Nglazi MD, Lawn SD, Kaplan R, Kranzer K, Orrell C, Wood R (2011). Changes in programmatic outcomes during 7 years of scale-up at a community-based antiretroviral treatment service in South Africa. J Acquir Immune Defic Syndr.

[32] Cornell M, Grimsrud A, Fairall L, Fox MP, van Cutsem G, Giddy J (2010). Temporal changes in programme outcomes among adult patients initiating antiretroviral therapy across South Africa, 2002–2007. AIDS.

[33] Grimsrud A, Balkan S, Casas EC, Lujan J, Van Cutsem G, Poulet E (2014). Outcomes of antiretroviral therapy over a 10-year period of expansion: a multicohort analysis of African and Asian HIV programs. J Acquir Immune Defic Syndr.

[34] Welaga P, Hosegood V, Weiner R, Hill C, Herbst K, Newell ML (2009). Coming home to die? The association between migration and mortality in rural South Africa. BMC Public Health.

[35] Kabudula C, Joubert J, Touane-Nkhasi M, Kahn K, Rao C, Gómez-Olivé FX (2014). Evaluation of record linkage of mortality data between a health and demographic surveillance system and national civil registration system in South Africa. Popul Health Metr.

[36] Darikwa TB, Dorrington R (2011). The level and trends of child mortality in South Africa, 1996–2006. Afr Popul Stud.

[37] Kerber KJ, Lawn JE, Johnson LF, Mahy M, Dorrington RE, Phillips H (2013). South African child deaths 1990–2011: have HIV services reversed the trend enough to meet Millennium Development Goal 4?. AIDS.

[38] Hargreaves JR, Bonell CP, Morison LA, Kim JC, Phetla G, Porter JD (2007). Explaining continued high HIV prevalence in South Africa: socioeconomic factors, HIV incidence and sexual behaviour change among a rural cohort, 2001–2004. AIDS.

[39] Bärnighausen T, Hosegood V, Timæus IM, Newell ML (2007). The socioeconomic determinants of HIV incidence: evidence from a longitudinal, population-based study in rural South Africa. AIDS.

[40] Johnson LF, Dorrington RE, Bradshaw D, du Plessis H, Makubalo L (2009). The effect of educational attainment and other factors on HIV risk in South African women: results from antenatal surveillance, 2000–2005. AIDS.

[41] McCarthy K, Chersich MF, Vearey J, Meyer-Rath G, Jaffer A, Simpwalo S (2009). Good treatment outcomes among foreigners receiving antiretroviral therapy in Johannesburg, South Africa. Int J STD AIDS.

[42] Boulle A, Schomaker M, May MT, Hogg RS, Shepherd BE, Monge S (2014). Mortality in patients with HIV-1 infection starting antiretroviral therapy in South Africa, Europe, or North America: a collaborative analysis of prospective studies. PLoS Med.

[43] Fairall LR, Bachmann MO, Louwagie GM, van Vuuren C, Chikobvu P, Steyn D (2008). Effectiveness of antiretroviral treatment in a South African program: a cohort study. Arch Int Med.

[44] Geng EH, Emenyonu N, Bwana MB, Glidden DV, Martin JN (2008). Sampling-based approach to determining outcomes of patients lost to follow-up in antiretroviral therapy scale-up programs in Africa. J Am Med Assoc.

[45] An MW, Frangakis CE, Musick BS, Yiannoutsos CT (2009). The need for double-sampling designs in survival studies: an application to monitor PEPFAR. Biometrics.

[46] Henriques J, Pujades-Rodriguez M, McGuire M, Szumilin E, Iwaz J, Etard JF (2012). Comparison of methods to correct survival estimates and survival regression analysis on a large HIV African cohort. PLoS One.

[47] Kiragga AN, Castelnuovo B, Musomba R, Levin J, Kambugu A, Manabe YC (2013). Comparison of methods for correction of mortality estimates for loss to follow-up after ART initiation: a case of the Infectious Diseases Institute, Uganda. PLoS One.

[48] Bisson GP, Gaolathe T, Gross R, Rollins C, Bellamy S, Mogorosi M (2008). Overestimates of survival after HAART: implications for global scale-up efforts. PLoS One.

[49] van Hest R, Grant A, Abubakar I (2011). Quality assessment of capture-recapture studies in resource-limited countries. Trop Med Int Health.

