# Supplementary material for: A comparison of death recording by health centres and civil registration in South Africans receiving antiretroviral treatment
Source: J Int AIDS Soc. 2015 Dec 16;18(1):20628. doi: 10.7448/IAS.18.1.20628 (PMC4684576; doi:10.7448/IAS.18.1.20628)
Supplement: A comparison of death recording by health centres and civil registration in South Africans receiving antiretroviral treatment [file JIAS-18-20628-s001.pdf]

Supplementary material

As there is a need for age- and sex-specific correction factors that can be applied to vital registration statistics, we fitted four different models to predict the fraction of deaths occurring at age x, in individuals of sex g (g=0 for females and 1 for males), that are recorded through the vital registration system, $c(x, g)$. In the first model, analogous to a logistic regression model in which age is assumed to have a linear effect on completeness, the function is of the form

$$c_1(x,g) = \frac{1}{1+\exp\left(-\alpha_1 - \beta_1 g - \gamma_1 x\right)} \ .$$

The second model is similar to the first, but assumes that the effect of age is linear on the log scale:

$$c_2(x,g) = \frac{1}{1+\exp\left(-\alpha_2 - \beta_2 g - \gamma_2 \ln(x+1)\right)}.$$

As the completeness of death reporting in infancy might be greater than that in older children in some contexts [1, 2], we consider an alternative model in which an additional parameter (δ) determines the 'excess' completeness in infancy:

$$c_3(x,g) = \frac{1}{1+\exp\left(-\alpha_3 - \beta_3 g - \gamma_3 \ln(x+1) - \delta_3 \exp(-x)\right)}.$$

We consider a fourth model in which the sex difference in completeness is assumed to be greatest at age 25:

$$c_4(x,g) = \frac{1}{1+\exp\left(-\alpha_4 - \beta_4 g \exp\left(-2\left(\ln\left(\frac{x+1}{25+1}\right)\right)^2\right) - \gamma_4 \ln(x+1) - \delta_4 \exp(-x)\right)}.$$

The four models were compared in terms of Bayesian Information Criterion (BIC), and the predicted completeness of vital registration for the country as a whole was calculated by applying the resulting correction factors ($1/c(x, g)$) to the recorded numbers of deaths in South Africa in 2010 [3]. The completeness estimates from the four models were averaged using Bayesian Model Averaging, assigning equal prior weights to the four models [4].

The estimates of the four models of vital registration completeness are summarized in Table S1, and the model estimates of the probability of death being recorded on the death register are shown in Figure S1. The first and second models have similar likelihood values although they produce very different estimates of the age pattern. The third model provides a substantially better fit to the data than the second model ($p = 0.007$), but under-estimates the fraction of male deaths that are recorded in childhood (Figure S1a). The fourth model provides the best fit to the data and the lowest BIC value, and estimates that the fraction of deaths in the first year of life that are recorded on the death register is around 88%, consistent with the data (14 out of 16 infant deaths, 88%). The Bayesian model average of

completeness, weighted according to the age and sex profile of South African deaths in 2010, is 94.3% (86.7% in children aged <15 and 95.3% for ages 15+).

Table S1: Estimates of models of vital registration completeness

	Model 1	Model 2	Model 3	Model 4	Weighted average
α_i (constant)	1.6999	0.4351	-0.5937	-0.6597	
β_i (male sex)	-0.4368	-0.3860	-0.4131	-0.5779	
γ_i (age effect)	0.03411	0.7007	0.9885	1.0143	
δ_i (infancy effect)	-	-	4.1755	3.7471	
Log likelihood	-1161.69	-1162.02	-1158.34	-1156.26	
BIC	2349.1	2349.7	2350.9	2346.8	
Model weight	0.004	0.110	0.003	0.883	
Average completeness*					
All ages	93.8%	90.1%	94.0%	94.4%	94.3%
Children (0-14)	81.9%	62.7%	85.7%	87.0%	86.7%
Adults (15+)	95.4%	94.9%	95.0%	95.3%	95.3%

* Calculated for the age and sex profile of deaths recorded in South Africa in 2010 [3].

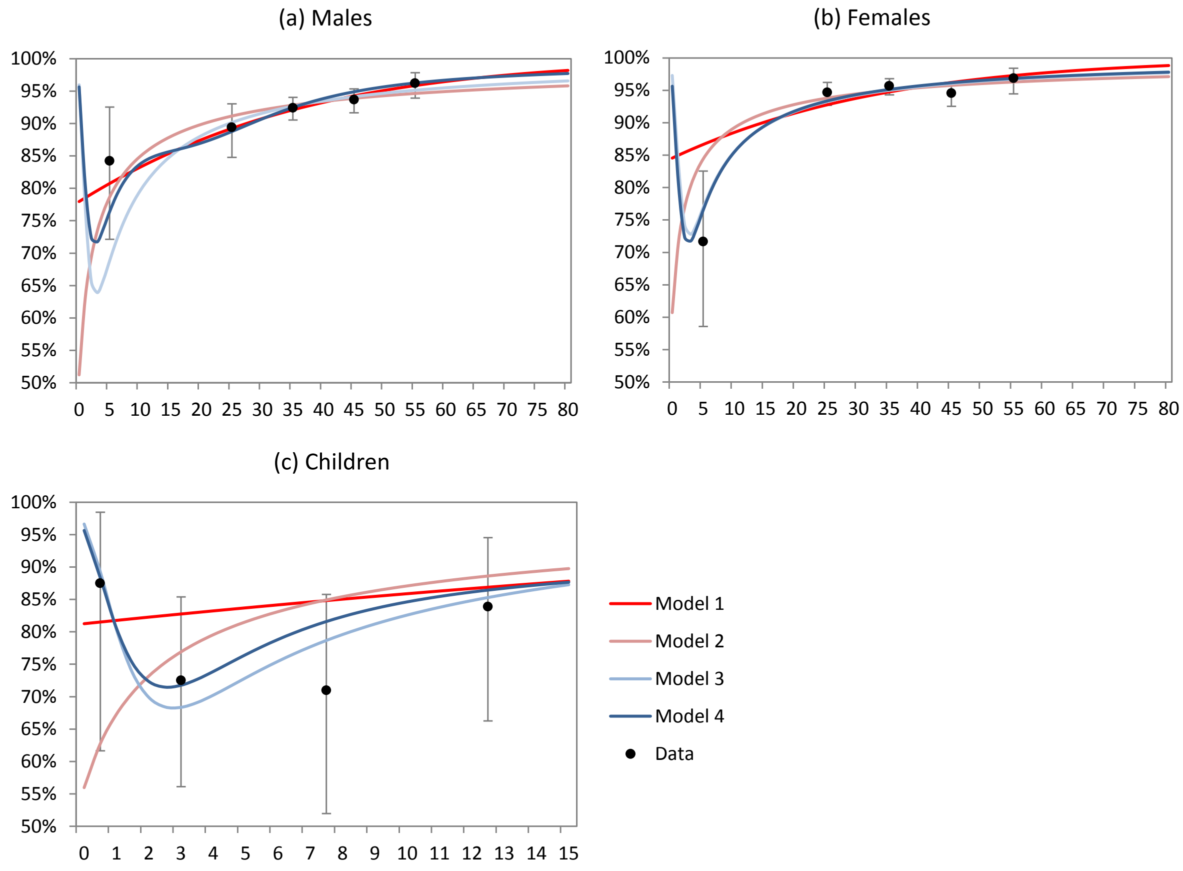

Figure S1: Fraction of deaths recorded on the death register

In panels (a) and (b), data plotted at ages 5, 25, 35, 45 and 55 represent the average proportions over the 0-14, 15-29, 30-39, 40-49 and 50+ age groups respectively. In panel (c), data plotted at ages 0.5, 3.0, 7.5 and 12.5 represent the average proportions over the <1, 1-4, 5-9 and 10-14 age groups respectively. Model estimates shown in panel (c) are the averages of the estimates for males and females.

References

1. Vapattanawong P, Prasartkul P. Under-registration of deaths in Thailand in 2005-2006: results of cross-matching data from two sources. *Bulletin of the World Health Organization* 2011; **89**:806-812.
2. Darikwa TB, Dorrington R. The level and trends of child mortality in South Africa, 1996-2006. *African Population Studies* 2011; **25**:159-172.
3. Statistics South Africa. Mortality and causes of death in South Africa, 2013: Findings from death notification. Pretoria; 2014. Available: http://beta2.statssa.gov.za/publications/P03093/P030932013.pdf. Accessed 3 May 2015
4. Hoeting JA, Madigan D, Raftery AE, Volinsky CT. Bayesian Model Averaging: A tutorial. *Statistical Science* 1999; **14**:382-417.
